# Prevalence and Predictors of Sub-Acute Phase Disability after Injury among Hospitalised and Non-Hospitalised Groups: A Longitudinal Cohort Study

**DOI:** 10.1371/journal.pone.0044909

**Published:** 2012-09-11

**Authors:** Sarah Derrett, Ari Samaranayaka, Suzanne Wilson, John Langley, Shanthi Ameratunga, Ian D. Cameron, Rebbecca Lilley, Emma Wyeth, Gabrielle Davie

**Affiliations:** 1 Injury Prevention Research Unit, Department of Preventive and Social Medicine, Dunedin School of Medicine, University of Otago, Dunedin, New Zealand; 2 School of Population Health, Faculty of Medical and Health Sciences, University of Auckland, Auckland, New Zealand; 3 Rehabilitation Studies Unit, Sydney Medical School, University of Sydney, Sydney, Australia; 4 Te Roopū Rangahau Hauora Māori a Ngāi Tahu (Ngāi Tahu Māori Health Research Unit), Department of Preventive and Social Medicine, Dunedin School of Medicine, University of Otago, Dunedin, New Zealand; Federal University of Rio de Janeiro, Brazil

## Abstract

**Introduction:**

To reduce the burden on injury survivors and their supporters, factors associated with poor outcomes need to be identified so that timely post-injury interventions can be implemented. To date, few studies have investigated outcomes for both those who were hospitalised and those who were not.

**Aim:**

To describe the prevalence and to identify pre-injury and injury-related predictors of disability among hospitalised and non-hospitalised people, three months after injury.

**Methods:**

Participants in the Prospective Outcomes of Injury Study were aged 18–64 years and on an injury entitlement claims register with New Zealand's no-fault injury compensation insurer, following referral by healthcare professionals. A wide range of pre-injury demographic, health and injury-related characteristics were collected at interview. Participants were categorised as ‘hospitalised’ if they were placed on New Zealand's National Minimum Data Set within seven days of the injury event. Injury severity scores (NISS) and 12 injury categories were derived from ICD-10 codes. WHODAS assessed disability. Multivariable analyses examined relationships between explanatory variables and disability.

**Results:**

Of 2856 participants, 2752 (96%) had WHODAS scores available for multivariable analysis; 673 were hospitalised; 2079 were not. Disability was highly prevalent among hospitalised (53.6%) and non-hospitalised (39.4%) participants, three-months after injury. In both groups, pre-injury disability, obesity and higher injury severity were associated with increased odds of post-injury disability. A range of other factors were associated with disability in only one group: e.g. female, ≥2 chronic conditions and leg fracture among hospitalised; aged 35–54 years, trouble accessing healthcare, spine or lower extremity sprains/dislocations and assault among non-hospitalised.

**Significance:**

Disability was highly prevalent among both groups yet, with a few exceptions, factors associated with disability were not common to both groups. Where possible, including a range of injured people in studies, hospitalised and not, will increase understanding of the burden of disability in the sub-acute phase.

## Introduction

For most countries, injury is the leading cause of mortality among younger people [1]. Injuries are also known to have important consequences for survivors of injury; particularly for those whose injury does not heal promptly and precludes return to pre-injury function, activity and participation in life [2], [3]. More needs to be known about the factors associated with poor outcomes at a population level [4]–[6]. In addition, to reduce burden among injury survivors and their families/supporters, characteristics associated with poor outcomes need to be identified so that timely and effective post-injury interventions can be implemented.

Despite the acknowledged gap in understanding the burden associated with poor outcomes following injury, few studies have investigated outcomes for people with ‘all types of injury’ [7], [8]. Furthermore, research considering ‘all injury’ has tended to focus on general health, functional or employment outcomes rather than disability [7], [9]–[12].

In studies of injury outcome, few have investigated outcomes for non-hospitalised people; perhaps because of an assumption that injured people who are not hospitalised are likely to have short recovery periods [13]. In the Netherlands, researchers found that people followed up after an emergency department (ED) visit for an injury had recovered, on a global measure of functional status, to a level equivalent to the general population, whereas people admitted to hospital for their injury had not [14]. However, a meta-analysis of data from studies of functioning after injury in a number of countries, found poor outcomes associated with injuries not commonly involving hospitalisation, such as sprains and strains [8]. A study of outcomes for survivors of car crashes found that among those with ‘minor’ injuries (Abbreviated Injury Scale; AIS = 1), as many as 10% had a permanent medical impairment [15]. The prospective UK Burden of Injury Study recruited injured participants from hospital in-patient admissions and emergency-department (ED) attendees [6]. This study found that disability was greater among in-patients yet, at the level of the population, considerable disability burden was estimated for those attending ED without a hospital admission [6].

**Table 1:** Pre-injury socio-demographic characteristics of participants according to hospitalisation status (n = 2752).

Explanatory variables		Hospitalised		Non-hospitalised		P value**
		n	%*	n	%*	
Age	18–34 years	266	27.9	689	72.1	0.007
	35–54 years	284	22.1	1002	77.9	
	55–64 years	123	24.1	388	75.9	
Gender	Male	458	27.1	1231	72.9	<0.001
	Female	215	20.2	848	79.8	
Education	None	106	25.4	311	74.6	0.909
	Secondary school	159	23.7	512	76.3	
	Other	397	24.5	1226	75.5	
Living arrangements	Alone	74	28.2	188	71.8	0.483
	With non-family	60	24.1	189	75.9	
	With family	537	24.1	1693	75.9	
Paid employment	Yes	610	24.1	1922	75.9	0.258
	No	63	28.8	156	71.2	
Financial status	Sufficient	604	24.5	1866	75.5	0.026
	Insufficient	57	22.3	199	77.7	

* Row percentage.

** P value from Chi2 test to compare hospitalised and non-hospitalised groups between variable categories.

**Table 2:** Pre-injury health and psychosocial characteristics of participants according to hospitalisation status (n = 2752).

Explanatory variables		Hospitalised		Non-hospitalised		P value**
		n	%*	n	%*	
General health	Excellent/ Very good/ Good	642	24.7	1960	75.3	0.540
	Fair/ Poor	30	20.7	115	79.3	
Chronic conditions	0	361	26.0	1025	74.0	0.046
	1	184	25.0	551	75.0	
	≥2	111	20.4	433	79.6	
Depressive-type episode	No	537	24.4	1663	75.6	0.895
	Yes	135	24.7	411	75.3	
Optimism	Yes	598	25.0	1798	75.0	0.192
	No	69	21.8	248	78.2	
Self-efficacy	Not poor	619	25.0	1853	75.0	0.085
	Poor	50	19.8	202	80.2	
Comfort in faith or spiritual beliefs	Very much/ Quite a bit	214	23.0	718	77.0	0.323
	Somewhat/ A little bit/ Not at all	435	25.4	1277	74.6	
	Missing	24	22.2	84	77.8	
Family involvement	Very large/ Large	586	24.2	1836	75.8	0.632
	Small/ Very small	83	26.6	229	73.4	
Social relationships	Satisfied	630	24.5	1945	75.5	0.852
	Not satisfied	39	23.8	125	76.2	
Sense of community	Strong	189	22.8	639	77.2	0.627
	In-between	282	25.2	839	74.8	
	Very little	169	25.3	499	74.7	
	Missing	33	24.4	102	75.6	
Physical activity	≥5 days	378	25.4	1109	74.6	0.440
	<5 days	282	23.3	926	76.7	
Sleep	≥5 nights	512	24.9	1543	75.1	0.557
	<5 nights	148	22.9	498	77.1	
BMI	Underweight/ Normal/ Overweight	477	24.2	1497	75.8	0.552
	Obese	162	24.6	497	75.4	
	Missing	34	28.6	85	71.4	
Smoking	No	465	24.2	1458	75.8	0.858
	Yes	206	25.1	616	74.9	
Alcohol use	Low	287	22.5	987	77.5	0.007
	Moderate	216	23.7	695	76.3	
	High	163	30.0	381	70.0	
Recreational drug use	No	524	23.6	1697	76.4	0.099
	Yes	147	28.1	377	71.9	

* Row percentage.

** P value from Chi2 test to compare hospitalised and non-hospitalised groups between variable categories.

**Table 3:** Injury-related characteristics of participants according to hospitalisation status (n = 2752).

Explanatory variables		Hospitalised		Non-hospitalised		P value**
		n	%*	n	%*	
Injury cause	Accidental	620	23.6	2010	76.4	<0.001
	Assault	51	46.4	59	53.6	
Threat to life	No	506	21.2	1881	78.8	<0.001
	Yes/ Maybe	144	44.7	178	55.3	
Threat of severe longer-term disability	No	312	19.8	1267	80.2	<0.001
	Yes/ Maybe	339	30.2	784	69.8	
Access to healthcare services	No trouble	586	23.9	1863	76.1	0.184
	Trouble/Mixed	80	28.9	197	71.1	
Injury severity	NISS 1–3	174	15.7	936	84.3	<0.001
	NISS 4–6	321	27.6	842	72.4	
	NISS >6	162	41.9	225	58.1	
*Injury categories*#						
Intracranial	No	624	23.5	2030	76.5	<0.001
	Yes	49	50.0	49	50.0	
Head/neck superficial	No	624	23.5	2030	76.5	<0.001
	Yes	49	50.0	49	50.0	
Spine sprain/ strain or dislocation	No	623	26.9	1691	73.1	<0.001
	Yes	50	11.4	388	88.6	
Upper extremity fracture	No	499	22.0	1771	78.0	<0.001
	Yes	174	36.1	308	63.9	
Upper extremity sprain/ strain or dislocation	No	602	25.5	1756	74.5	0.001
	Yes	71	18.0	323	82.0	
Upper extremity open wound	No	597	23.1	1992	76.9	<0.001
	Yes	76	46.6	87	53.4	
Upper extremity superficial	No	642	24.5	1978	75.5	0.790
	Yes	31	23.5	101	76.5	
Lower extremity fracture	No	474	20.8	1810	79.2	<0.001
	Yes	199	42.5	269	57.5	
Lower extremity sprain/ strain or dislocation	No	583	28.2	1486	71.8	<0.001
	Yes	90	13.2	593	86.8	
Lower extremity open wound	No	624	23.6	2018	76.4	<0.001
	Yes	49	44.5	61	55.5	
Lower extremity superficial	No	638	24.8	1932	75.2	0.090
	Yes	35	19.2	147	80.8	
Other	No	472	20.7	1803	79.3	<0.001
	Yes	201	42.1	276	57.9	

* Row percentage.

** P value from Chi2 test to compare hospitalised and non-hospitalised groups between variable categories.

# Multiple injury categories possible.

**Table 4:** Prevalence (and 95%CI) of participants with WHODAS disability scores ≥10 according to pre-injury socio-demographic characteristics and hospitalisation status (n = 2752).

Explanatory variables		Hospitalised			Non-hospitalised		
		%	95% CI		%	95% CI	
Age	18–34 years	52.6	46.6	58.4	34.7	31.1	38.2
	35–54 years	53.2	47.4	59.0	43.4	40.3	46.5
	55–64 years	56.9	48.1	65.7	37.1	32.3	41.9
Gender	Male	49.6	45.0	54.1	36.4	33.7	39.1
	Female	62.3	55.8	68.8	43.6	40.2	47.0
Education	None	53.8	44.2	63.3	45.7	40.1	51.2
	Secondary school	49.7	41.9	57.5	36.3	32.2	40.5
	Other	55.4	50.5	60.3	39.1	36.3	41.8
Living arrangements	Alone	48.7	37.2	60.1	37.2	30.3	44.2
	With non-family	60.0	47.5	72.5	36.5	29.6	43.4
	With family	53.5	49.2	57.7	39.9	37.6	42.3
Paid employment	Yes	52.8	48.8	56.8	39.0	36.8	41.2
	No	61.9	49.8	74.0	43.0	35.2	50.7
Financial status	Sufficient	53.8	49.8	57.8	38.3	36.1	40.5
	Insufficient	54.4	41.3	67.4	48.7	41.8	55.7

**Table 5:** Prevalence (and 95%CI) of participants with WHODAS disability scores ≥10 according to pre-injury health and psychosocial characteristics and hospitalisation status (n = 2752).

Explanatory variables		Hospitalised			Non-hospitalised		
		%	95% CI		%	95% CI	
General health	Excellent/ Very good/ Good	53.6	49.6	57.5	38.9	36.7	41.1
	Fair/ Poor	53.3	34.3	71.7	46.1	36.7	55.6
Chronic conditions	0	49.9	45.0	55.0	35.8	32.9	38.7
	1	52.2	44.9	59.4	38.8	34.8	42.9
	≥2	68.5	59.8	77.2	48.7	44.0	53.4
Depressive-type episode	No	51.8	47.5	56.0	38.0	35.7	40.3
	Yes	60.7	52.5	69.0	45.3	40.4	50.1
Optimism	Yes	53.3	49.3	57.3	38.9	36.7	41.2
	No	58.0	46.2	69.7	44.4	38.2	50.6
Self-efficacy	Not poor	52.8	48.9	56.8	38.8	36.6	41.0
	Poor	66.0	52.7	79.3	45.5	38.6	52.4
Comfort in faith or spiritual beliefs	Very much/ Quite a bit	62.6	55.8	69.1	43.7	40.1	47.4
	Somewhat/ A little bit/ Not at all	49.9	45.1	54.6	37.5	34.8	40.2
	Missing	41.7	22.1	63.4	29.8	20.2	40.7
Family involvement	Very large/ Large	54.1	50.1	58.1	38.8	36.5	41.0
	Small/ Very small	50.6	39.8	61.4	43.2	36.8	49.7
Social relationships	Satisfied	52.9	49.0	56.8	38.9	36.7	41.0
	Not satisfied	61.5	46.1	77.0	46.4	37.6	55.2
Sense of community	Strong	53.4	46.3	60.6	39.3	35.5	43.1
	In-between	51.4	45.6	57.3	38.3	35.0	41.6
	Very little	57.4	49.9	64.9	40.3	36.0	44.6
	Missing	54.6	37.3	71.8	44.1	34.4	53.8
Physical activity	≥5 days	55.6	50.5	60.6	40.5	37.6	43.4
	<5 days	50.7	44.9	56.6	38.1	35.0	41.3
Sleep	≥5 nights	52.9	48.6	57.3	40.4	38.0	42.9
	<5 nights	56.8	48.7	64.8	35.9	31.7	40.2
BMI	Underweight/ Normal/ Overweight	50.1	45.6	54.6	36.3	33.9	38.8
	Obese	63.6	56.1	71.0	47.9	43.5	52.3
	Missing	55.9	38.9	72.8	42.4	31.8	52.9
Smoking	No	56.3	51.8	60.9	37.9	35.4	40.4
	Yes	48.1	41.2	54.9	43.0	39.1	46.9
Alcohol use	Low	56.5	50.7	62.2	40.5	37.4	43.6
	Moderate	53.2	46.6	59.9	38.1	34.5	41.7
	High	50.3	42.6	58.0	38.3	33.4	43.2
Recreational drug use	No	54.2	49.8	58.5	40.2	37.4	42.6
	Yes	52.4	44.0	60.7	35.5	30.7	40.6

**Table 6:** Prevalence (and 95%CI) of participants with WHODAS disability scores ≥10 according to injury-related characteristics and hospitalisation status (n = 2752).

Explanatory variables		Hospitalised			Non-hospitalised		
		%	95% CI		%	95% CI	
Injury cause	Accidental	53.6	49.6	57.5	38.8	36.7	40.9
	Assault	52.9	39.1	66.8	55.9	43.1	68.7
Threat to life	No	50.4	46.0	54.8	38.0	35.8	40.2
	Yes/ Maybe	59.0	51.0	67.1	52.8	45.5	60.2
Threat of severe longer-term disability	No	45.5	40.0	51.0	35.8	33.2	38.5
	Yes/ Maybe	59.0	53.8	64.2	44.1	40.7	47.6
Access to healthcare services	No trouble	53.4	49.4	57.5	37.8	35.6	40.0
	Trouble/ Mixed	55.0	44.0	66.0	52.3	45.3	59.3
Injury severity	NISS 1–3	35.6	28.5	42.8	34.8	31.8	37.9
	NISS 4–6	57.0	51.6	62.4	40.4	37.1	43.7
	NISS >6	65.4	58.1	72.8	52.9	46.3	59.4
*Injury categories*#							
Intracranial	No	53.2	49.3	57.1	39.2	37.0	41.3
	Yes	59.2	45.3	73.1	46.9	32.8	61.1
Head/neck superficial	No	53.0	49.1	57.0	39.4	37.3	41.5
	Yes	61.2	47.4	75.0	36.7	23.1	50.4
Spine sprain/ strain or dislocation	No	52.5	48.6	56.4	36.7	34.4	38.9
	Yes	68.0	54.9	81.1	51.0	46.0	56.0
Upper extremity fracture	No	56.9	52.6	61.3	40.4	38.1	42.7
	Yes	44.3	36.8	51.7	33.1	27.9	38.4
Upper extremity sprain/ strain or dislocation	No	55.3	51.3	59.3	39.9	37.6	42.2
	Yes	39.4	28.0	51.0	36.5	31.3	41.8
Upper extremity open wound	No	55.4	51.4	59.4	40.0	37.8	42.1
	Yes	39.5	28.4	50.5	25.3	16.1	34.5
Upper extremity superficial	No	52.5	48.6	56.4	39.5	37.4	41.7
	Yes	77.4	62.5	92.4	35.6	26.3	45.0
Lower extremity fracture	No	44.9	40.5	49.4	39.6	37.4	41.9
	Yes	74.4	68.3	80.5	37.6	31.7	43.3
Lower extremity sprain/ strain or dislocation	No	51.6	47.6	55.7	37.4	34.9	39.8
	Yes	66.7	56.9	76.5	44.4	40.3	48.4
Lower extremity open wound	No	54.2	50.3	58.1	39.6	37.5	41.8
	Yes	46.9	32.8	61.1	29.5	18.0	41.0
Lower extremity superficial	No	53.6	49.7	57.5	39.2	37.0	41.4
	Yes	54.3	37.5	71.0	41.5	33.5	49.5
Other	No	52.3	47.8	56.8	39.5	37.2	41.7
	Yes	56.7	49.8	63.6	38.4	32.7	44.2

# Multiple injury categories possible.

**Table 7:** Multivariable analyses of pre-injury and injury-related characteristics associated with disability three months after the injury event for each sub-group, hospitalised (n = 590) and non-hospitalised (n = 1838).

Explanatory variables		Hospitalised			Non-hospitalised		
		OR	95% CI		OR	95% CI	
Pre-injury WHODAS disability score		1.13	1.04	1.23	1.17	1.13	1.22
Age	18–34 years	1.00	reference				
	35–54 years	0.87	0.57	1.34	1.40	1.11	1.78
	55–64 years	0.94	0.52	1.69	1.04	0.76	1.43
Gender	Male	1.00	reference				
	Female	1.78	1.14	2.80	1.23	0.99	1.52
Financial status	Sufficient	1.00	reference				
	Insufficient	1.14	0.55	2.34	1.51	1.06	2.14
General health	Excellent/ Very good/ Good	1.00	reference				
	Fair/Poor	0.39	0.15	1.02	0.61	0.36	1.01
Chronic conditions	0	1.00	reference				
	1	1.14	0.72	1.79	1.02	0.79	1.30
	≥2	1.92	1.06	3.46	1.17	0.88	1.56
Comfort in faith or spiritual beliefs	Very much/ Quite a bit	1.00	reference				
	Somewhat/ A little bit/ Not at all	0.53	0.34	0.83	0.75	0.60	0.93
	Missing	1.15	0.29	4.63	0.84	0.45	1.57
Physical activity	≥5 days	1.00	reference				
	<5 days	0.79	0.54	1.18	0.79	0.64	0.97
Sleep	≥5 nights	1.00	reference				
	<5 nights	0.95	0.59	1.53	0.70	0.55	0.90
BMI	Underweight/ Normal/ Overweight	1.00	reference				
	Obese	1.93	1.19	3.13	1.49	1.17	1.89
	Missing	0.92	0.34	2.48	0.66	0.35	1.25
Injury cause	Accidental	1.00	reference				
	Assault	1.49	0.71	3.12	3.04	1.54	6.01
Threat to life	No	1.00	reference				
	Yes/ Maybe	0.92	0.55	1.53	1.52	1.02	2.26
Threat of severe longer-term disability	No	1.00	reference				
	Yes/ Maybe	1.61	1.07	2.41	1.20	0.96	1.49
Access to healthcare services	No trouble	1.00	reference				
	Trouble/ Mixed	0.72	0.39	1.33	1.92	1.37	2.69
Injury severity	NISS 1–3	1.00	reference				
	NISS 4–6	2.17	1.23	3.82	1.65	1.23	2.20
	NISS >6	2.69	1.30	5.56	2.01	1.40	2.88
*Injury categories#*							
Intracranial		0.57	0.22	1.50	0.89	0.41	1.92
Head/neck superficial		1.96	0.85	4.49	0.65	0.27	1.57
Spine sprain/ strain or dislocation		1.94	0.89	4.22	2.21	1.57	3.11
Upper extremity fracture		0.57	0.32	1.01	0.78	0.53	1.15
Upper extremity sprain/ strain or dislocation		0.64	0.33	1.25	1.14	0.82	1.59
Upper extremity open wound		0.96	0.49	1.87	0.62	0.34	1.14
Upper extremity superficial		2.23	0.77	6.46	1.11	0.67	1.83
Lower extremity fracture		3.50	1.91	6.42	1.09	0.74	1.60
Lower extremity sprain/ strain or dislocation		1.75	0.91	3.37	1.64	1.19	2.28
Lower extremity open wound		0.70	0.32	1.51	0.70	0.35	1.41
Lower extremity superficial		0.88	0.36	2.14	1.25	0.83	1.88
Other		1.47	0.88	2.46	1.00	0.69	1.45

# Each of the injury category odds ratios are with reference to those not having an injury of that category.

To date, we do not know about the risk of adverse disability outcomes for those who are not hospitalised following injury. The Prospective Outcomes of Injury Study (POIS) provides an ideal opportunity to investigate factors associated with disability for both hospitalised and non-hospitalised injured people.

POIS is a longitudinal cohort study in New Zealand with the primary objective of identifying factors associated with disability following injury. The aims of this paper are threefold: 1) To determine whether there are differences in pre-injury and injury-related characteristics between hospitalised and non-hospitalised sub-groups of POIS participants; 2) To describe the prevalence of disability in the sub-acute phase, three months after injury, for both hospitalised and non-hospitalised groups; and 3) To identify pre-injury and injury-related predictors of disability among hospitalised and non-hospitalised sub-groups.

## Methods

### Study Participants

The study was undertaken following approval from the New Zealand Health and Disability Multi-Region Ethics Committee (MEC/07/07/093). Following feedback from participants in the pilot study and to be inclusive of all people (including those with poor vision or limited literacy), and with the approval of the Ethics Committee, all participants granted oral consent to participate after receiving comprehensive information about the study. Oral consent was documented by interviewers, and all participants received copies of the consent form. POIS design, recruitment and participants' characteristics have been described previously [16], [17]. Briefly, potential participants were aged 18–64 years and lived in one of five regions in New Zealand. Following referral by an accredited primary or secondary healthcare professional, participants had all been placed onto an injury (entitlement claims) register at the Accident Compensation Corporation (ACC), the organisation responsible for New Zealand's no-fault compensation scheme. All injured people in New Zealand are eligible for accident compensation by law; approximately 1.75 million injury claims are lodged with ACC annually; ‘entitlement claimants’ comprise approximately 7% of injuries referred to ACC [18], [19]. These people have injuries serious enough to potentially require ‘on-going support’, such as income compensation (if in paid employment), medical treatment costs and social and vocational services [19]. People were not eligible for the study if they had been placed on the sensitive claims register (e.g. people who have been sexually assaulted) or if their injury was the result of self-harm [17].

Between December 2007 and August 2009, 7875 entitlement claimants were sent letters about the study by ACC, of whom 4881 people were able to be contacted by the research team; 2856 (59%) participated in the first interview three months (on average) after injury [17].

### Outcome

The measure of disability was the brief World Health Organization Disability Assessment Schedule II 12-item instrument (WHODAS) [20]. This assesses activity limitations and participation restrictions over the past 30 days along six dimensions (understanding and communication, self-care, mobility, interpersonal relationships, work and household roles, and community roles), with 12 questions (concerning standing, household responsibilities, learning, community activities, emotionally affected by health problems, concentrating, walking a long distance, washing whole body, getting dressed, dealing with unknown people, friendships, day to day work). Each of the twelve questions has five difficulty-level response options: ‘None = 0, Mild = 1, Moderate = 2, Severe = 3 or Extreme/Cannot do = 4’. The 12 response scores were summed using the simple-summed approach to provide a total score with a possible range from 0 (no disability) to 48 (maximum disability) [21]. Participants missing a response to one of the 12 items had their average score, from the 11 completed responses, imputed for the missing response; if more than one response was missing, the participant's scores were not summed for analysis [21]. Participants were asked to report WHODAS status in the 30 days pre-injury and in the 30 days before the interview (three months after injury). For multivariable analysis, participants were dichotomised into ‘disability’ or ‘no disability’ groups based on whether their WHODAS score was greater than, or equal to, 10 [21].

### Hospital

Participants in this study were categorised as ‘hospitalised’ if they appeared in New Zealand's National Minimum Data Set (NMDS) which: “For the purposes of the national collections, healthcare users who receive assessment and/or treatment for three hours or more, or who have a general anaesthetic, are to be admitted. This also applies to healthcare users of Emergency Departments” [22]. To identify those admitted in proximal response to the injury, a threshold of admission within seven days from the date of the injury event was used [23]. POIS data were probabilistically linked to NMDS data to identify hospitalised participants.

### Explanatory variables

Explanatory variables were grouped according to pre-injury socio-demographic, pre-injury health and psychosocial and injury-related characteristics.

#### Pre-injury socio-demographic characteristics

Participants self-reported their socio-demographic characteristics including age at time of interview, gender, education and living arrangements, based on questions from the New Zealand Census [24]. Education was grouped as ‘none’, ‘secondary school’ (i.e. high school) level or ‘other’ qualifications (if these took three months or more to obtain). ‘Living arrangements’ were grouped as ‘alone’, with ‘non-family’ or with ‘family’ (including partner or spouse). People working full-time (≥30 hours per week) or part-time (<30 hours per week) were classified as being in ‘paid employment’; the remaining as ‘not in paid employment’ [25]. ‘Financial status’ was classified as ‘sufficient’ if participants reported they had ‘just enough, enough or more than enough’ total household income to meet their every day needs; and ‘insufficient’ if they reported ‘not enough’ pre-injury [25].

#### Pre-injury health and psychosocial characteristics

Participants rated their pre-injury ‘general health’ on a five-point scale (‘excellent’, ‘very good’, ‘good’, ‘fair’ or ‘poor’) [26]. Pre-injury ‘chronic conditions’ was assessed using questions modified from the New Zealand Health Survey 2006/2007 [27]. Participants reported whether they had been told by a doctor that they had one or more of a list of 22 chronic illnesses or diseases (e.g. asthma, cancer, diabetes, depression or anxiety) that had lasted, or was expected to last, for more than six months.

Participants were classified as having a ‘depressive-type episode’ if they responded affirmatively to at least one of two screening questions asking whether nearly every day, for a period of two weeks or more in the year before injury, they had felt ‘sad, blue or depressed’, and/or ‘loss of interest in things like work or hobbies or things they usually like to do for fun’ [28]. Pre-injury ‘optimism’ was measured by asking a single question from the Life Orientation Test [29]. Participants who ‘strongly agreed’ or ‘agreed’ with the statement that “Overall, I expect more good things to happen to me than bad” were compared with the rest. ‘Self-efficacy’ was based on the General Self-Efficacy Scale, a scale that assesses problem-solving capabilities in relation to a variety of difficult demands in ten aspects of life [30]. The responses ‘strongly disagree’, ‘disagree’, ‘neutral/mixed’, ‘agree’, and ‘strongly agree’ were scored from 0 to 4 respectively and summed to provide a total score. Poor self-efficacy was defined as a score ≤25, out of a possible score of 40. Participants were asked if they found “comfort in faith and spiritual beliefs”, using a single question from the FACIT-Sp (permission granted by www.facit.org), which had five options ranging from ‘not at all’ to ‘very much’ [31].

‘Family involvement’ was assessed by asking whether family played a ‘very large’, ‘large’, ‘small’ or ‘very small’ part in participants’ lives pre-injury [25]. Participants rated their overall satisfaction with ‘social relationships’ (including contact with relatives and friends, quality of relationships with your partner and/or family and frequency of social contact). Those stating they were ‘completely’ or ‘mostly’ satisfied were classified as ‘satisfied’; those stating they were ‘neither satisfied nor dissatisfied’, ‘mostly’ or ‘completely’ dissatisfied were classified as ‘not satisfied’. People stated whether they felt their neighbourhood’s ‘sense of community’ was ‘strong’, ‘very little’ or ‘something in-between’ [32].

Participants reported pre-injury ‘physical activity’ according to the number of days in the seven-day period prior to injury that they had engaged in either 30 minutes of moderate activity (including brisk walking) or 15 minutes of vigorous activity [33]. Pre-injury ‘sleep’ was assessed by asking participants to report the number of nights per week that they (usually) had seven or more hours sleep. Participants reported pre-injury height and weight; derived Body Mass Index (‘BMI’) categories were underweight (BMI<18.5), normal (18.5–24.9), overweight (25–29.9) and obese (≥30), with the first three combined for analyses [34]. Pre-injury ‘smoking’ was determined by asking whether or not people smoke cigarettes regularly [35]. The brief Alcohol Use Disorders Identification Test (AUDIT-C) was used to categorise participants into three ‘alcohol use’ groups according to their drinking patterns in the year before injury: low (males AUDIT-C score 0–4; females 0–3), moderate (males 5–7; females 4–6) or high (males 8–12; females 7–12) [36]. Participants were asked about the frequency of ‘recreational drug use’, i.e. marijuana/cannabis and other recreational drugs such as methamphetamine, speed, ecstasy, LSD or cocaine, with responses being: ‘never’, ‘monthly or less’, ‘2–4 times a month’, ‘2–3 times a week’, ‘4 or more times a week’. Those who responded they never used substances in the year before injury were classified as ‘no’.

#### Injury characteristics

At interview, participants were asked to report if the ‘injury cause’ was accidental or a physical assault; whether, at the time of injury, they felt the injury was a ‘threat to their life’; and whether they felt the injury was a ‘threat of severe longer-term disability’. For each of these questions those responding ‘yes’ or ‘maybe/possibly’ were grouped together and compared with those responding ‘no’. Information about ‘access to healthcare services’ was obtained by asking people if they had trouble getting to or contacting health services; ‘yes’ or ‘mixed’ were grouped together and compared with those who said ‘no’.

All injury diagnosis information for each participant was collected from ACC in the form of Read, International Classification of Diseases ICD-9 or ICD-10 codes allocated by healthcare professionals [37]. To ensure all codes were in the same format, Read codes were mapped to ICD-10 (3-character level) using a mapping file provided by ACC; ICD-9 codes were mapped to ICD-10 using publicly-available mapping files from New Zealand's Ministry of Health. Of the 4794 ICD-10 codes thus obtained, 405 were either not within, or did not map to, S or T injury codes. Typically, these were injury sequelae (e.g. a diagnosis of cellulitis secondary to the injury) or medical procedures. These diagnoses were recoded to the index injury identified from the text accompanying the ACC diagnosis or from participants' own descriptions of their injury. Injuries without an S or T code were manually recoded (e.g. hernia was recoded to ‘other injury of abdomen’) to fit within the ‘diagnosis and body part’ matrix described below. There were 112 S and T codes which did not specify either the injury or the part of the body (e.g. unspecified injury of lower leg, fracture of unspecified body region); these were also recoded to fit the matrix based on participants' own descriptions of their injury. If the specific index injury was coded elsewhere in the diagnosis list, the sequelae, procedure or non-specific codes were not used.

As all diagnoses were collected, participants could have more than one injury type (e.g. both a fracture and a sprain) or more than one part of their body injured (e.g. both an arm and a leg). Twelve injury categories were developed using the ICD-10 codes to describe both the injured body region and nature of injury, based on the ICD-10 injury mortality diagnosis matrix and the Barell injury diagnosis matrix [38], [39]. The 12 injury categories were: intracranial, head/neck superficial, spine sprain/strain or dislocation, upper extremity fracture, upper extremity sprain/strain or dislocation, upper extremity open wound, upper extremity superficial, lower extremity fracture, lower extremity sprain/strain or dislocation, lower extremity open wound, lower extremity superficial, and other anatomical region/nature. ‘Upper extremity’ includes the shoulder; ‘lower extremity’ the hip. The first 11 injury categories comprise all those containing more than 100 cases; all remaining injuries (e.g. crush, amputation, burn) were collapsed into the heterogeneous ‘other’ category. For analysis, all participants were classified according to whether or not they had sustained an injury in any of the 12 categories following the same approach used by Holtslag et al [40].

A New Injury Severity Score (NISS) was also derived for each participant [41]. ICD-10 codes were converted to AIS scores using a computer program [42]. Codes which did not map to an AIS score using the program were reviewed using the AIS manual [43]. Where an AIS score could not be derived for all ICD-10 codes of a participant, that person was considered missing for the purposes of calculating NISS. Potential AIS scores range from 1 (minor) to 6 (maximum (currently untreatable)) [44]. NISS was calculated as the sum of the squares of an individual's three highest AIS scores (or all their AIS scores if they have fewer than four diagnoses) [41], and grouped for analysis into three severity categories: 1–3 (least severe; AIS = 1 injuries only), 4–6 (middle severity; one AIS = 2 injury plus none, one or two AIS = 1 injuries) and >6 (most severe; at least two AIS = 2 injuries or one AIS≥3 injury) [41].

### Analysis

Bivariate analyses (chi-squared tests) were completed to compare the proportions of participants hospitalised and non-hospitalised according to each of the explanatory variables. Proportions reporting WHODAS disability three months after injury are presented with 95% confidence intervals (95%CI). Discussion of bivariate results is focused on results with p-values of less than 0.01 or discrete (non-overlapping) 95%CI.

For multivariable analyses exploring relationships between explanatory variables and WHODAS disability for the hospitalised and non-hospitalised groups, a two-step process was used. First, two separate multivariable logistic regression models were built for each of the hospitalised and non-hospitalised groups using a stepwise backward selection procedure with a threshold p-value of ≤0.1. All explanatory variables listed in Tables 1, 2, 3 were considered for inclusion in each of these two independent models. When more than 100 responses were missing for any explanatory variable (e.g. sense of community), a separate category was presented and labelled ‘missing’ to allow participants missing such variables to be included in the model. If ≤100 responses were missing for any variable, participants with missing data for that variable were not included in this initial model-building (results not presented).

Second, all variables retained in either the hospitalised and non-hospitalised groups' independent models were entered into two further ‘consistent variable’ models. This allows us to present the odds ratio (OR), and 95%CI, of WHODAS disability after injury consistently for both groups. These two models include all cases with non-missing responses in retained variables (subjected to the above ≤100 criterion). In all four models, ‘time between the injury event and interview’ was adjusted for, as this was known to vary between participants and also to be associated with disability. Age, gender, NISS and the 12 injury categories were also retained in all models. Stata 11.1 was used for analysis [45].

## Results

Of 2856 POIS participants, 104 were missing either a pre-injury or three-month post-injury WHODAS score, leaving data from 2752 (96%) available for analysis.

### Bivariate analyses

Tables 1, 2, 3 present the proportions of the hospitalised (n = 673; 24.5%) and non-hospitalised (n = 2079; 75.5%) groups according to each of the listed variables. Of the pre-injury socio-demographic characteristics (Table 1), a greater proportion of people in the youngest age group (18–34 years) and males were treated in hospital as a consequence of their injury; conversely, people in the middle age group (35–54 years) were less likely to be hospitalised. Considering pre-injury health and psychosocial characteristics (Table 2), a greater proportion of those with high alcohol use were hospitalised. Among injury-related variables (Table 3), a greater proportion of those reporting their injury resulted from assault and was a threat to their life or of disability, and those with NISS of four or more, were hospitalised; NISS in this study ranged from 1 to 22. For the 12 injury categories a greater proportion of those with intracranial injury, head or neck superficial injuries, extremity fractures, open wounds, and ‘other’ injuries were hospitalised. Conversely, a smaller proportion of those with sprains, strains or dislocations were hospitalised. No differences in proportions were apparent for either upper or lower extremity superficial injury.

Before injury, the prevalence of disability (WHODAS summed score ≥10) was 4.5% for the hospitalised group and 5.3% for the non-hospitalised group. Tables 4, 5, 6 present the prevalence of disability three months after the injury event, according to each explanatory variable. Overall, sub-acute phase disability was more prevalent among injured participants treated at hospital (53.6%) than those not. However, among the non-hospitalised group disability was experienced by more than one-third (39.4%) three months after the injury event.

### Multivariable analyses

Table 7 presents the multivariable models for the hospitalised and non-hospitalised groups. After applying the ‘≤100 missing’ restriction described above, data were missing for at least one of the independent variables (presented in Tables 1, 2, 3) for 324 participants; including 92 who were missing a NISS score, one of the variables deliberately retained in each model. Consequently, data from 590 hospitalised and 1838 non-hospitalised participants were available for multivariable analysis. P-values for Hosmer-Lemeshow goodness-of-fit for the hospitalised and non-hospitalised models are acceptable; p = 0.17 and 0.64 respectively. The area under the curve for each model indicates reasonable disability discrimination: 80.0% and 72.5% respectively.

#### Pre-injury disability

In both the hospitalised and non-hospitalised groups, pre-injury disability was associated with increased odds of post-injury disability. For every point increase in pre-injury WHODAS score, there was a 13% increase in odds of disability among the hospitalised and a 17% increase among the non-hospitalised.

#### Pre-injury socio-demographic characteristics

Among the hospitalised group, age did not appear to be associated with the odds of disability. However, among the non-hospitalised group being in the middle age group (35–54 years) was associated with increased disability (OR = 1.40). In the hospitalised model, but not the non-hospitalised, being female increases the odds of disability after injury (OR = 1.78). Having insufficient finances pre-injury was associated with increased disability among the non-hospitalised group only (OR = 1.51). Other socio-demographic characteristics (education, living arrangements and paid employment) were not retained in either model.

#### Pre-injury health and psychosocial characteristics

Having two or more chronic conditions resulted in nearly twice the odds of disability for the hospitalised group only (compared to those with no chronic conditions; OR = 1.92). Reporting less comfort in faith or spiritual beliefs was associated with lower odds of disability among both groups (compared to those with quite a bit or very much comfort; OR = 0.53 for hospitalised and 0.75 for non-hospitalised). In the non-hospitalised group only, compared to the relevant reference categories, not engaging in regular physical activity and not having enough sleep pre-injury reduced the odds of disability (OR = 0.79 and 0.70 respectively). Being obese was associated with disability among both the hospitalised and non-hospitalised groups (compared to non-obese; OR = 1.93 for hospitalised; OR = 1.49 for non-hospitalised).

A number of pre-injury health and psychosocial variables were not retained in the models; namely pre-injury general health, depressive-type episodes in the year before injury, optimism, self-efficacy, family involvement, social relationship satisfaction, sense of community, smoking, alcohol use and recreational drug use.

#### Injury-related characteristics

A three-fold increased odds of disability was experienced by non-hospitalised participants reporting their injury to be the result of an assault (OR = 3.04) compared to those reporting an unintentional injury. The non-hospitalised group were also at increased odds when they perceived a threat to their life (OR = 1.52) and the hospitalised group when they perceived a threat of longer-term disability (OR = 1.61). Trouble accessing health services among the non-hospitalised group was associated with nearly twice the odds of disability (OR = 1.92).

In both the hospitalised and non-hospitalised groups having a NISS score of either 4–6 or >6 (compared to those with scores of 1–3; OR = 2.17 and 2.69 respectively for hospitalised, and 1.65 and 2.01 for non-hospitalised) is independently associated with disability after injury. However, among the 12 injury categories, only three were independently associated with disability. A 3.5-fold increased odds of disability is experienced by hospitalised people with a lower extremity fracture (OR = 3.50) compared to those without such an injury. Having a spine or lower extremity sprain/strain or dislocation (OR = 2.21 and 1.64 respectively) was associated with increased odds of disability in the non-hospitalised group.

## Discussion

We compared a wide range of pre-injury and injury-related characteristics among people with injuries, including both those who were hospitalised as a consequence of their injury (n = 673) and those who were not (n = 2079). Bivariate analyses revealed few differences between the hospitalised and non-hospitalised groups in pre-injury socio-demographic, health and psychosocial characteristics; more variation was apparent among the injury-related characteristics (Tables 1, 2, 3). Disability was experienced by 53.6% of the hospitalised group three months, on average, after injury. It is noteworthy that more than one-third of the people not hospitalised (39.4%) were also experiencing disability at this time.

Pre-existing disability is a strong and independent predictor of disability in the sub-acute post-injury period for both those treated at hospital and those not (Table 7). Analysis of data from the large National Health Interview Survey in the United States found that adults with pre-existing disability had poorer access to health care, and calls were made for removal of barriers to access for people with disability [46]. In our study all participants had to have had at least some contact with health professionals to become registered with ACC and thereby eligible for possible recruitment to POIS. Assessment of pre-injury disability could be useful in effectively targeting people at risk of poor outcome for a more comprehensive and/or tailored package of treatment and rehabilitation services.

Aside from pre-injury disability, a number of other characteristics were independently associated with disability three months after injury for the hospitalised group (Table 7). These included being female, having two or more pre-existing chronic conditions, obesity, perceiving a threat of disability, a NISS score >3 and a lower extremity fracture. We, and others, have previously found being female places people at increased risk of other types of poor outcome [9], [47], [48]. However, a smaller Norwegian study of longer-term disability outcomes among people with NISS>15, did not find an association between WHODAS (36-item version) and gender [49]. Worse outcomes may be associated with poorer care being provided to women [48], [50].

For the non-hospitalised group, in contrast to the hospitalised group, being female, having pre-existing chronic conditions or a perceived threat of disability were not independently associated with disability. Further research is required to understand why these independent relationships were not observed among our non-hospitalised participants while accounting for a wide range of other characteristics including age, NISS and injury category. Associations with increased odds of disability in the sub-acute phase are only similar between the hospitalised and non-hospitalised groups for pre-injury disability, obesity and NISS.

More associations were apparent in the model for the non-hospitalised group than the hospitalised group, although the smaller size of the hospitalised group may have led to insufficient power to detect associations that do, in fact, exist. Among the non-hospitalised, people aged 35–54 years were at increased odds of disability, as were those with insufficient finances, injury due to assault, perceived threat to their life, trouble accessing healthcare services and those with spine or lower extremity sprains/strains or dislocations. Of particular note were people reporting assault as the cause of their injury. This group were at three-fold increased odds of disability compared to those not reporting assault, after accounting for other factors including injury severity. To our knowledge, this is the first study to examine the relationship between assault and disability in a non-hospitalised injured population. Further research needs to be undertaken to understand the reason for the increased odds of disability. For example, it is plausible that such poor odds among the non-hospitalised may arise as a consequence of appropriate support being less available to the non-hospitalised assaulted group than to those who were assaulted but treated at hospital.

Certain characteristics were associated with lower odds of disability. Among both the hospitalised and non-hospitalised groups, having little comfort in faith or spiritual beliefs reduced the odds of disability. Faith and spirituality are complex concepts [51]. While acknowledging that ‘religion’ is not the same concept as ‘faith’, certain religious practices may have a positive relationship with health or provide a sense of meaning when adverse health events occur (consolation model) [51]. Conversely, for some people adverse health events may be interpreted as a punishment by God (punishment model) [51], [52]. In their study investigating relationships between the FACIT-Sp-12 and health outcomes reported by cancer survivors, Edmondson et al found the two components of the FACIT, religious and existential well-being, were conceptually distinct with different effects on cancer survivors' health [53]. Existential well-being was more strongly associated with health outcomes than was religious well-being [53]. The single question used in our study is from the religious well-being component, which may explain our result. We encourage others to include a wider range of spirituality questions in studies of outcome following injury to confirm, or otherwise, the association between injury and disability outcome.

Counter-intuitively, not engaging in regular pre-injury physical activity or having adequate sleep were also associated with lower odds of disability among the non-hospitalised group only. Previously, we have found similar (protective) associations between low levels of pre-injury physical activity and functional and work outcomes [48], [54]. We wonder if such an association indicates that those who were not exercising or sleeping well pre-injury are not suffering as much from their loss post-injury; in contrast to those who were exercising and sleeping well before injury. These findings may also have arisen by chance. Future analyses will examine whether or not these associations continue into the longer-term.

In summary, we found a wide range of pre-injury demographic, health and injury-related characteristics associated with increased odds of disability in the sub-acute phase. It is interesting to observe that of the 12 injury categories only three (lower extremity fracture for hospitalised; spine or lower extremity sprain/strains/dislocations for the non-hospitalised) are independently associated with increased odds of disability outcomes; whereas overall injury severity (NISS) is a consistent predictor of outcome among both the hospitalised and non-hospitalised groups. With a few exceptions, the characteristics associated with increased odds of disability are not consistent between the hospitalised and non-hospitalised groups. This indicates that caution should be used in generalising results from studies of hospitalised patients to people with injuries not resulting in hospital treatment.

### Strengths and limitations

A strength of our study is the inclusion of both people who were hospitalised and people who were not. Further, many injury outcome studies have focused on very specific injury types (e.g. traumatic brain injury or hip fracture) or causes (e.g., falls, road crashes), rather than ‘all injury’ types as we have done. Another strength of our study is that we were able to include injury severity, and a comprehensive range of pre-injury and injury-related factors.

Our study has some limitations. First, the fact that participants were asked to recall pre-injury characteristics three months after injury introduced the possibility of recall bias. Although most pre-injury factors (e.g. level of education, living arrangements) did not rely on ‘subjective’ ratings, the measure of pre-injury disability did. In a study considering other health status outcomes, researchers have found peoples' recalled pre-injury health status was (re-)attained when they also reported having recovered, suggesting reasonable recall of pre-injury states [55]. However, their study was investigating recall using a different measure to the WHODAS and we cannot discount some bias in relation to recall of pre-injury disability. Despite this limitation, it is a strength that we have used the WHODAS, an instrument specifically developed to assess disability, as an outcome measure [56], [57]. Few injury studies have considered disability as an outcome; and fewer still have used validated measures of disability [49], [58], [59]. The WHODAS was developed by the World Health Organization in conjunction with advisors, including groups of people experiencing disability [21], [60].

Our classification of hospitalisation excludes cases who sought treatment or assessment at hospital, but whose visit lasted less than three hours, because these are not required to be reported to NMDS. People with injuries that typically require treatment in the acute phase only, without the need for more than a week off work, or on-going treatment, were also excluded from our study [13]. Another limitation is that few people in our cohort had injuries with very high severity scores (highest NISS being 22). Consequently, although a strength of our study is that we have been able to include people with (all) injuries whether or not they resulted in hospitalisation, it also means caution should be used when interpreting our odds of disability for those with NISS >6. A study in Denmark found no association between higher injury severity (ISS) categories and health-related quality of life outcomes among injured participants with ISS≥9 [61]. Similar research is required to understand relationships between higher NISS and disability. Furthermore, although the inclusion of a wide range of pre-injury characteristics was a strength of our study, this also led to a limitation. To minimise burden to participants it was not possible to include lengthy sets of questions about every characteristic included. We did not find associations between pre-injury psychological variables and disability outcomes, or between a number of other characteristics, such as smoking and alcohol use, and disability using the measures employed in the POIS study. Future analyses of data collected at subsequent POIS follow-up points (12- and 24-months) will ascertain whether relationships exist between psychosocial characteristics present three months after injury and longer-term poor outcomes, as others have found [59], [62].

### Conclusions

Our study is one of a small, but growing, number to report disability outcomes following all-injury using a measure developed specifically for disability, the WHODAS. Our study suggests that it would be unwise to generalise results from the hospitalised population to the non-hospitalised population. The UK Burden of Injury Study estimated considerable collective burden following injury for people seen at ED but not hospitalised, in part because of the greater numbers with injuries not resulting in hospitalisation [6]. Our study suggests that a considerable proportion of non-hospitalised individuals carry a significant disability burden following injury. It would be desirable for other studies, where possible, to investigate outcomes following injury for those who are not hospitalised. Elsewhere, others have called for longer-term assessment of outcome following injury [63]. Our research group is now analysing disability (and other outcomes) to 12-month and 24-month follow-up points, and will also examine trajectories of recovery (or not) over time, as undertaken in a smaller New Zealand study examining health status following car crashes [64].
